# Optimising EEG-fMRI for Localisation of Focal Epilepsy in Children

**DOI:** 10.1371/journal.pone.0149048

**Published:** 2016-02-12

**Authors:** Maria Centeno, Tim M. Tierney, Suejen Perani, Elhum A. Shamshiri, Kelly StPier, Charlotte Wilkinson, Daniel Konn, Tina Banks, Serge Vulliemoz, Louis Lemieux, Ronit M. Pressler, Christopher A. Clark, J. Helen Cross, David W Carmichael

**Affiliations:** 1 Developmental imaging and biophysics Section, Institute of child health, University College London, London, United Kingdom; 2 Epilepsy Unit, Great Ormond Street Hospital, London, United Kingdom; 3 Division of Neuroscience, Institute of Psychiatry, Psychology & Neuroscience, King's College London, London, United Kingdom; 4 Neurophysiology Department, University Hospital Southampton, Southampton, United Kingdom; 5 EEG and Epilepsy Unit, Neurology, University Hospitals and Faculty of Medicine of Geneva, Geneva, Switzerland; 6 Department of Clinical and Experimental epilepsy, Institute of Neurology, University College London, London, United Kingdom; University of Electronic Science and Technology of China, CHINA

## Abstract

**Background:**

Early surgical intervention in children with drug resistant epilepsy has benefits but requires using tolerable and minimally invasive tests. EEG-fMRI studies have demonstrated good sensitivity for the localization of epileptic focus but a poor yield although the reasons for this have not been systematically addressed. While adults EEG-fMRI studies are performed in the “resting state”; children are commonly sedated however, this has associated risks and potential confounds. In this study, we assessed the impact of the following factors on the tolerability and results of EEG-fMRI in children: viewing a movie inside the scanner; movement; occurrence of interictal epileptiform discharges (IED); scan duration and design efficiency. This work’s motivation is to optimize EEG-fMRI parameters to make this test widely available to paediatric population

**Methods:**

Forty-six children with focal epilepsy and 20 controls (6–18) underwent EEG-fMRI. For two 10 minutes sessions subjects were told to lie still with eyes closed, as it is classically performed in adult studies (“rest sessions”), for another two sessions, subjects watched a child friendly stimulation i.e. movie (“movie sessions”). IED were mapped with EEG-fMRI for each session and across sessions. The resulting maps were classified as concordant/discordant with the presumed epileptogenic focus for each subject.

**Findings:**

Movement increased with scan duration, but the movie reduced movement by ~40% when played within the first 20 minutes. There was no effect of movie on the occurrence of IED, nor in the concordance of the test. Ability of EEG-fMRI to map the epileptogenic region was similar for the 20 and 40 minute scan durations. Design efficiency was predictive of concordance.

**Conclusions:**

A child friendly natural stimulus improves the tolerability of EEG-fMRI and reduces in-scanner movement without having an effect on IED occurrence and quality of EEG-fMRI maps. This allowed us to scan children as young as 6 and obtain localising information without sedation. Our data suggest that ~20 minutes is the optimal length of scanning for EEG-fMRI studies in children with frequent IED. The efficiency of the fMRI design derived from spontaneous IED generation is an important factor for producing concordant results.

## Introduction

EEG-fMRI has shown over the last decade to be a useful technique to map epileptic networks across a range of epilepsy syndromes [1–3]. It provides clinically useful information for the localization of the epileptic focus [4–7] and may have a role in the prediction of surgical outcome [6, 8, 9] of drug resistant focal epilepsies. Furthermore, it has played a key role in the characterization of epileptic networks in focal and generalized epilepsies [4, 10, 11] as well as revealing the involvement of resting state networks during epileptic activity [4, 10, 12]. Recent studies [13–15] have shown that network dynamics can be investigated with EEG-fMRI, offering opportunities to further understanding the pathophysiology of epileptic activity.

However EEG-fMRI studies have a number of potential limitations that may play a role in the sensitivity of this test to map the epileptogenic region. These include:

1. Low yield: Due to the unpredictable nature of IED [6]
2. Long duration: In order to maximize the chances of capturing IED EEG-fMRI studies tend to use long scanning times. The optimal length for EEG-fMRI studies has not been formally assessed.
3. Lack of motion control: associated with long scanning times
4. Variability and poor definition of baseline. Differently from cognitive fMRI studies, EEG-fMRI baseline is unconstrained and this may affect the power of the test.

There are no studies systematically addressing these interacting factors that affect EEG-fMRI sensitivity and tolerability

A large proportion of the epileptic syndromes develop during infancy and childhood [16]. From the therapeutic point of view, recent studies suggest that epilepsy surgery may relate to better outcome in terms of seizure freedom and cognitive abilities when performed at an earlier stage [17–19]. EEG epileptiform discharges (IEDs) are found in children with epilepsy during routine EEGs with rates between 18% and 56%[20], IEDs are also more frequent if seizures are ongoing. Therefore in children with focal epilepsy with poor seizure control it is more likely to capture IED during an EEG-fMRI scan. These considerations suggest that EEG-fMRI may have a large yield in children with epilepsy and could contribute to the assessment of surgery in a population that can be highly benefited from it. Therefore it is timely that EEG-fMRI is made available to paediatric population.

One of the main potential limitations of MRI in a paediatric population in general is the effects of movement on data quality and the tolerance of children to the test. Adult’s long resting state EEG-fMRI protocols are unlikely to be widely tolerable in a paediatric population. Most previous EEG-fMRI studies in children have used sedation to control movement inside the scanner [21, 22]. However, the effect of sedation on epileptic activity and related-hemodynamic changes remain poorly understood. Furthermore, imaging under sedation has a number of additional problems: it increases the scanning complexity (due to the need for close medical supervision); there is a risk (albeit low) of medical complications; it limits the extrapolation of methods that have been clinically validated in adult studies, where sedation is not used; it makes group studies more difficult for the following reasons: not all patients may require sedation, and levels of sedation may vary between patients and comparison with healthy control subjects is not possible, as using sedation is rarely ethically possible in control subjects.

Natural stimulation has been proposed as a way to improve compliance and reduce movement as well as to create a more comfortable experience in the scanner for paediatric populations [23]. Natural stimulation aims to provide sensory (audio-visual) stimulation whilst minimally interfering with cognitive networks, provided that this stimulation has a low cognitive demand. Preliminary reports have suggested that resting state network study results can be replicated when applying low level sensory stimulation such as movies or visual-auditory paradigms [24].

The effects of low level sensory stimulation such as a movies on epileptic activity are unknown as is the impact on the functional maps derived from EEG-fMRI studies. To our knowledge, no previous EEG-fMRI study has used nor investigated this approach.

In this study, we aim to test the impact of: a) a child friendly natural stimulus (i.e. movie); b) in-scanner head movement; c)the occurrence of interictal epileptiform discharges (IED); d) scan duration; e) design efficiency; on the results of EEG-fMRI for IED localisation in children with drug-resistant focal epilepsy.

## Material and Methods

The study was approved by NRES Committee London—Surrey Borders Research Ethics Committee (REC) London Centre. REC reference: 11/LO/1421 Written informed consent was obtained from all the participants’ guardians in the participant’s behalf.

### 2.1 Subjects

46 children (25 female; age: mean 13.7 y) with drug resistant epilepsy underwent simultaneous EEG-fMRI.

Recruited patients were undergoing evaluation for resective epilepsy surgery at Great Ormond Street Hospital (London, UK). Inclusion criteria for the study were: the presence of frequent interictal epileptiform discharges on EEG and participant ages between 6 and 18. Additionally, a group of 20 healthy volunteers (14 female) with an age between 6.6–16.7 years and a mean age of 11.5 years were scanned following the same protocol.

Controls were significantly younger than patients in our population. We controlled for this effect statistically when investigating the effect of natural stimulation on in-scanner movement by using age as a confound.

#### 2.1.1 Clinical assessment of the epileptogenic region

All patients underwent detailed clinical evaluation, prolonged video telemetry-EEG to document seizures and structural MRI. Additional tests including FDG-PET, ictal SPECT, MEG and intracranial EEG were performed in those patients in whom additional information for the localisation of the epileptogenic region was required. The localization of the epileptogenic region was determined by consensus between a group of expert epileptologists based on the review of the results of the panel of diagnostic tests. Clinical discussion to determine the epileptogenic region takes place at a multidisciplinary meeting that included neurology, neurosurgery, neurophysiology, neuroradiology and neuropsychology specialists. See Table 1 for clinical information

**Table 1:** Clinical and EEG-fMRI information: Summary of each case’s clinical and radiological information. The presumed epileptogenic region is established during the presurgical evaluation process for each patient after evaluation of a panel of tests. N IED types is the number of different IED identified in each subject and IED/ session shows the mean number of IED in each session. EEG interictal epileptiform discharges (IED) column shows the field of the epileptiform activity captured during EEG-fMRI scan. EEG-fMRI map shows the classification of the maps: concordant or discordant. Ep. Region: epileptogenic F: Female; M: male: L left; R: right; FCD: focal cortical dysplasia. N/A: non applicable. T-P-O: Temporo-parieto-occipital C: Concordant EEG-fMRI map with epileptogenic region, D: Discordant EEG-fMRI map with epileptogenic region.

ID	Age/ Gender	Presumed ep. region	MRI lesion/location	NIED types	IED/session	EEG IED	BOLD in ep. region	BOLD outside ep. region	EEG-fMRI map classification
**# 1**	8F	L Temporal	Tuberosclerosis. Left temporal prominent tuber	1	5	L temporal	N	N	D
**# 2**	14F	L Frontal pole	N/A			No IED			N/A
**# 3**	11M	Hypothalamus/L Temporal	Hypothalamic hamartoma	1	34.5	Left temporal	N	Y	D
**# 4**	15M	L posterior quadrant	N/A			No IED			N/A
**# 5**	11F	R Fronto-temporal	N/A	1	40.5	R Fronto-temporal	N	N	D
**# 6**	17M	R Parietal	R parietal FCD. Residual lesion after surgery	4	167	R Temporal	Y	N	C
**# 7**	15M	R Frontal-central	N/A			No IED			N/A
**# 8**	15M	L Temporal/R Frontal	L Temporal lobe FCD	3	219	R Frontal	Y	Y	C
**# 9**	11F	L Frontal-central	L hemisphere cortical atrophy perinatal	1	237.6	L central	Y	N	C
**# 10**	14F	R Temporal	R temporal FCD 2B	2	169	R Temporal	Y	Y	C
**# 11**	11F	R Fronto-temporal	N/A	1	134.7	R Fronto-temporal	Y	Y	C
**# 12**	11F	R Frontal pole	R frontal pole FCD	1	129.5	R Fronto-polar	Y	Y	C
**# 13**	12M	R central	Skull malformation/ R hemispheric cortical atrophy	3	151	R central	N	Y	D
**# 14**	17F	R Frontal	N/A	1	50	R Frontal	Y	Y	C
**# 15**	16F	L Frontal	N/A	1	10.5	Bifrontal spike-wave runs	Y	Y	C
**# 16**	11F	L Frontal	N/A	1	20	L Frontal lateral	N	Y	D
**# 17**	14M	L Fronto-temporal	L hemisphere cortical atrophy Autoinmune	2	187.2	L Temporal	Y	Y	C
**# 18**	16F	L posterior insula	L insular FCD	1	66.7	L central	N	N	D
**# 19**	18M	R parietal superior and medial	R precuneus FCD 2A	1	44.7	R Parietal	N	Y	D
**# 20**	11M	R Frontal	N/A	2	151.2	R Frontal	Y	Y	C
**# 21**	16F	L posterior quadrant/L frontal	L T-P-O polymicrogyria /Unspecific cortical atrophy	3	261.5	L posterior quadrant	Y	Y	C
**# 22**	15M	L Temporo-occipital	L HS	2	101	L posterior quadrant	Y	Y	C
**# 23**	17F	L Temporal	N/A			No IED			N/A
**# 24**	17F	L Frontal	N/A			No IED			N/A
**# 25**	8F	L Frontal	L middle cerebral artery stroke	1	107	Bifrontal spike-wave runs	Y	Y	C
**# 26**	16M	R Frontal	N/A	2	114.2	R Frontal	N	N	D
**# 27**	11M	L posterior quadrant	L choroid plexus papilloma L posterior quadrant atrophy secondary to radiotherapy	2	294	L posterior quadrant	N	N	D
**# 28**	13M	L Frontal	L middle frontal gyrus FCD	1	29	L Frontal	N	N	D
**# 29**	10F	L Frontal	N/A	2	87.5	L Frontal	N	N	D
**# 30**	11M	R posterior quadrant	N/A	3	53.5	R posterior quadrant	Y	Y	C
**# 31**	14M	L Occipital	L posterior artery stroke	1	57.5	L Occipital	Y	Y	C
**# 32**	17M	L Frontal	N/A			No IED			N/A
**# 33**	17M	R Occipital	L occipital atrophy. Iquemic perinatal insult	1	40.25	L Occipital	Y	Y	C
**# 34**	13M	L Occipital	L T-P-O cortical atrophy perinatal	3	64	L posterior quadrant	Y	Y	C
**# 35**	18F	R Frontal	Right frontal FCD			No IED			N/A
**# 36**	17F	R Frontal/R temporal	Bilateral oribito-frontal polymicrogyria	2	219	R Temporal	Y	Y	C
**# 37**	18F	R F-T-P junction	R T-P-O junction postusurgery residual FCD			No IED			N/A
**# 38**	11F	R Parietal	N/A	1	77.5	R central	N	N	D
**# 39**	18M	R Parietal	R inferior parietal residual postsurgical DNET	2	94.5	R Parietal	N	Y	D
**# 40**	11F	R Parietal	R parietal FCD	1	375.75	R Parietal	Y	Y	C
**# 41**	13F	R medial Temporal	R amygdala DNET	1	126.75	R anterior Temporal	Y	Y	C
**# 42**	12M	R Perisylvian	Bilateral perisylvian polymicrogyria	1	13.75	R posterior Fronto-Parietal	N	Y	D
**# 43**	17F	L Frontal	N/A	2	78.7	L Frontal-vertex	Y	Y	C
**# 44**	13F	L Frontal	L inferior frontal FCD	2	135.5	L Frontal lateral	Y	Y	C
**# 45**	15F	R posterior cingualte	R posterior cingulate DNET	1	47	R Frontal	N	Y	D
**# 46**	7M	L Frontal	N/A	1	74.5	L Frontal vertex	Y	Y	C

### 2.2 EEG-fMRI acquisition

#### 2.2.1 EEG-fMRI protocol

Participants were fitted with a 64-electrode MRI-compatible EEG cap (Easy cap, Brain Products, Munich, Germany). Preparation commenced 1 hour before the scanning time. Prior to placing the subject in the MRI scanner, scalp EEG was recorded for 10 minutes to obtain a baseline EEG.

Subjects were then transferred to the MRI scanner table. The head was immobilized using a vacuum and foam cushions. Subjects watched a TV screen via a mirror mounted on the head coil and sound was delivered through a set of MRI compatible headphones which supressed scanner acquisition noise.

The subjects were videoed inside the scanner with an MRI compatible camera (Nordic NeuroLabs, Bergen, Norway) interfaced with Brain Products recorder software.

The MRI protocol consisted of a T1-weighted volume scan followed by 4 10-minute fMRI sessions. For two of the fMRI sessions, the subjects were presented with a movie (a ‘Tom and Jerry’ cartoon clip) chosen to attract and maintain their attention (‘movie session’) and for the other two sessions, the subjects were instructed to rest with their eyes closed (‘rest session’). The movie presentation was configured as a paradigm with two blocks of cartoon lasting for 4 minutes each interleaved with 1.2 minute blocks where the screen displayed the words “please wait”. Rest and movie sessions were alternated and the participants were randomly allocated to start with the cartoon or resting session.

To investigate the tolerance of children to EEG-fMRI, subjects were asked after each session whether they were comfortable and if they would like to proceed with another session until they completed a maximum of 4 sessions.

Tolerance of our study population to EEG-fMRI is expressed as the fraction of patients that completed a certain proportion of the full protocol (measured as the number of completed sessions). Correlation between the age of the subject and the number of sessions tolerated was investigated by comparing the mean ages of subjects completing or failing to complete the full protocol using a T-test. Statistical tests were calculated using SPSS software version 21.

#### 2.2.2 MRI acquisition

Subjects were scanned in a 1.5T Siemens Avanto scanner at Great Ormond Street Hospital (London, UK) with a 12 channel receive head coil. Echo planar imaging (EPI) parameters are as follows: 3.3x3.3x4mm resolution with a field of view (FOV) = 210mm, TR = 2160ms, TE = 30ms, flip angle = 75 degrees, number of slices = 30, slice thickness = 3mm, slice gap = 1mm, and matrix 64x64, with 300 volumes and a voxel size of 3.3 x 3.3x 4.0 mm. Image acquisition was continuous.

T1-weighted whole-brain structural images were also obtained in all subjects using a Fast Low Angle Single Shot (FLASH) gradient echo sequence.

#### 2.2.3. EEG acquisition

EEG data was acquired with a 64 electrode MRI-compatible equipment (Easy cap and Brain Amp, Brain Products, Munich, Germany) simultaneously with MRI. Data was band-pass filtered at 0.016Hz-1 kHz with 16-bit digitalization (0.5μV resolution) and sampling rate was set to 5 kHz.

### 2.3 EEG pre-processing and analysis

MR gradient and pulse-related artefacts were removed from the EEG using template artefact subtraction [25, 26] implemented in a commercial EEG processing package (Brain Analyzer; Brain Products). EEG was down sampled to 250 Hz and filtered between 0.5 and 70 Hz for visual review. Interictal epileptiform activity was identified and manually marked on the EEG traces for each session by consensus between a clinical neurologist (MC) and one of the three neurophysiologists that participated in the study (KS,CW,DK).

Each of the EEGs was reviewed in both bipolar and referential montages for the accurate identification of epileptiform abnormalities.

### 2.4 EEG-fMRI analysis

EEG-fMRI analysis to determine the presence of regional IED-related BOLD changes was performed only for the patients and not for the controls.

The fMRI time series were analysed using a general linear model in SPM8 (http://www.fil.ion.ucl.ac.uk/spm/spm8). For this purpose, IEDs onsets and durations were converted into a temporal regressor, and convolved with the canonical hemodynamic response function (HRF) and its temporal and dispersion derivatives, resulting in 3 regressors for each event type [6].

MRI pre-processing consisted of volume-volume realignment and using SPM followed by pre-processing with FIACH [27] which removes non-physiological signal changes and creates a model of physiological noise. Slice timing correction and smoothing with an 8mm kernel was applied to the FIACH-processed images. Images were analysed in the subject’s space.

The six realignment parameters and six FIACH noise regressors were entered as regressors of no interest. An additional regressor with the paradigm waveform convolved with the HRF was entered for the movie sessions to account for the main effect of the task.

Effects related to IEDs were tested with an F-test across the three IED regressors (HRF and the two derivatives). Changes in BOLD signal were considered significant above a threshold with p<0.001 (uncorrected) and a cluster size voxel with a minimum of 5 contiguous voxels [5].

#### 2.4.1 Classification of EEG-fMRI maps

**Fig 1:**
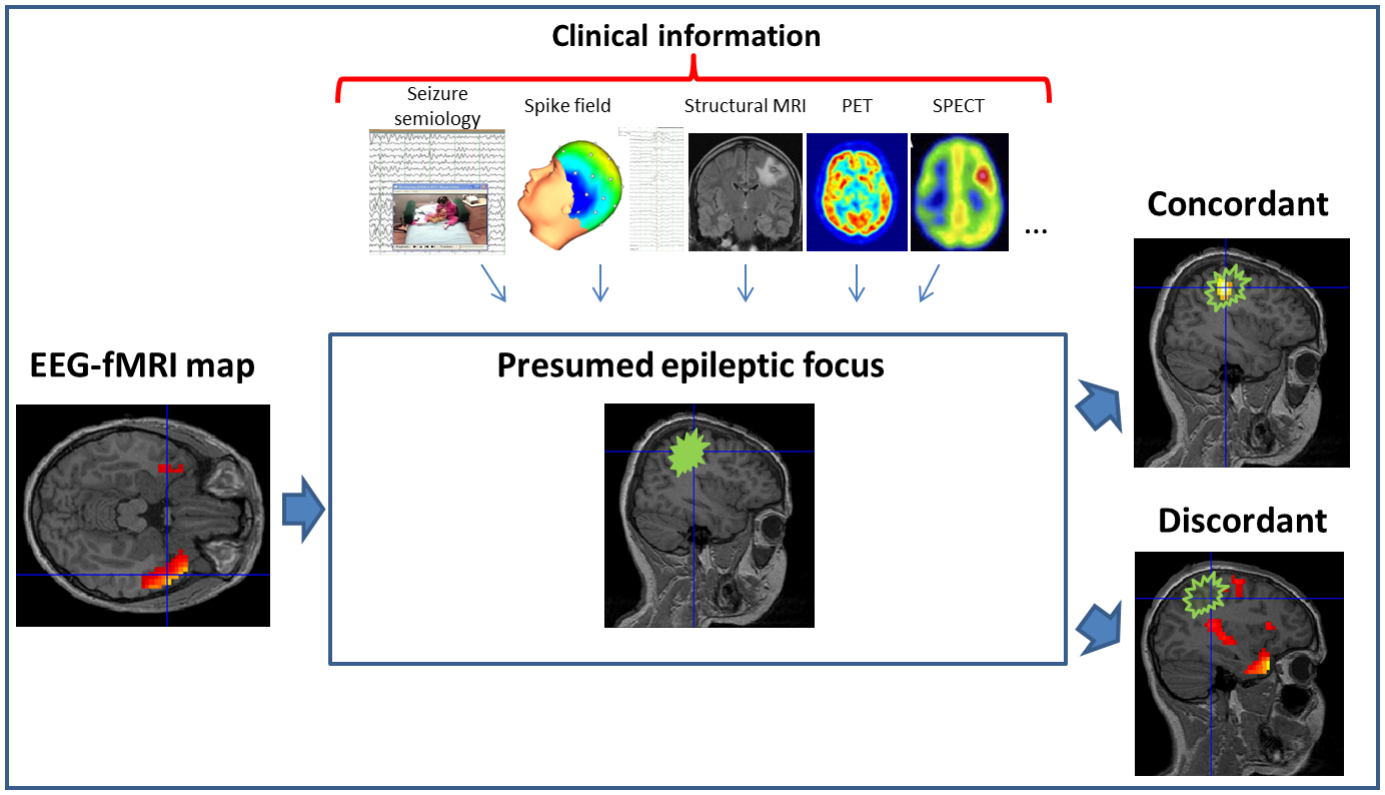
Clinical assessment of the EEG-fMRI maps. EEG-fMRI maps are compared with the location of the presumed epileptogenic region (green area). Epileptogenic region is assessed clinically based on multimodal tests. Maps are classified as Concordant if a cluster of significant activation is present in the epileptogenic region and Discordant otherwise.

EEG-fMRI maps were classified as concordant if they contained a cluster of significant BOLD signal change within the presumed epileptogenic region and discordant otherwise [4, 5, 28]. For those patients with multiple types of IED, maps were considered concordant if a cluster of significant BOLD signal changes was present within the presumed epileptogenic region for at least one of the IED types (Fig 1).

### 2.5 Analysis of movie effects

We analysed the effect of movie on the following variables:

#### 2.5.1 Effect of the movie on in-scanner movement

The effect of the movie on subject movement was estimated using a regression model. The dependant variable was movement inside the scanner which was quantified by mean frame-wise displacement (MFD) which is the difference in the position between each volume measured by spatial realignment in spm8 (see above). The MFD was transformed using the natural logarithm to normalize the distribution of the residuals. The independent variables were the session type (movie, rest), time (session order 1 to 4) and age. The interaction between session type and time was also explored.

The model parameters were estimated using a mixed effects model in R [29]. This model was chosen to account for the variability in baseline subject movement and the heterogeneous effect of duration on subject movement. As such the effect of time on movement was modelled as a random effect across subjects.

#### 2.5.2 Effect of movie on IED rate and on the concordance of EEG-fMRI maps

We compared the rate of IEDs during the movie and the rest sessions across all the patients. We further divided movie sessions into the different blocks of the paradigm (cartoon and “please wait” blocks) to account for potential differences in IED rate during the stimulus period. An ANOVA was used to assess the impact of condition on the occurrence of IEDs

EEG-fMRI maps were created for each session and the concordance of maps was assessed as detailed above. The effect of movie on the concordance of EEG-fMRI maps was tested using a two tailed chi square test of session type (movie/rest)*concordance of EEG-fMRI maps (SPSS version 21).

### 2.6 Effect of length of scanning on EEG-fMRI maps

To explore the effect of EEG-fMRI scan duration in our population, we calculated the proportion of maps with concordant/discordant results obtained when the first session was considered alone and compared this proportion to the concordance rate obtained when 2 sessions (20 minutes), three sessions (30 minutes) and four sessions (40 minutes) were considered. For this purpose, individual general lineal models were generated with either one; two; three or four sessions for each subject using spm8. Models were otherwise specified as described in the EEG-fMRI analysis section above.

### 2.7 Effect of Design Efficiency on EEG fMRI map quality

IED frequency and distribution should affect the sensitivity of EEG-fMRI to detect IED-related fMRI responses, and therefore explain the results in terms of concordance (here discordance includes a null result). It is necessary to quantify this effect in terms of the efficiency of the model to detect fMRI changes, that can be estimated as for cognitive fMRI paradigms using the design efficiency [30] which essentially measures the variability induced in the BOLD signal by the observed IEDs. We then used a logistic regression to determine whether efficiency can predict the concordance.

## Results

### 3.1 Tolerance of EEG-fMRI in paediatric population

68 children were recruited, of which 66 (46 patients and 20 controls) underwent EEG-fMRI. 83.3% (55/66) of them completed the whole protocol (4 sessions), while 13.7% (9/66) and 3% (2/66) completed 3 and 2 sessions respectively. One of the participants only tolerated the scan when the movie was being displayed.

There were 2 failed scans; one due to intolerance of the EEG-cap application and the second case due to fear of lying down in the scanner table and entering the scanner bore.

Using a two sample t-test it was found there was a statistically significant difference between the age of the patients and the number of sessions that they tolerated. Patients who completed the 4 sessions had a mean age of 13.7 years compared to 9.6 years for those not completing the protocol (t(44) = 3.457, p< 0.05).

### 3.2 Effect of the movie on in-scanner movement

**Fig 2:**
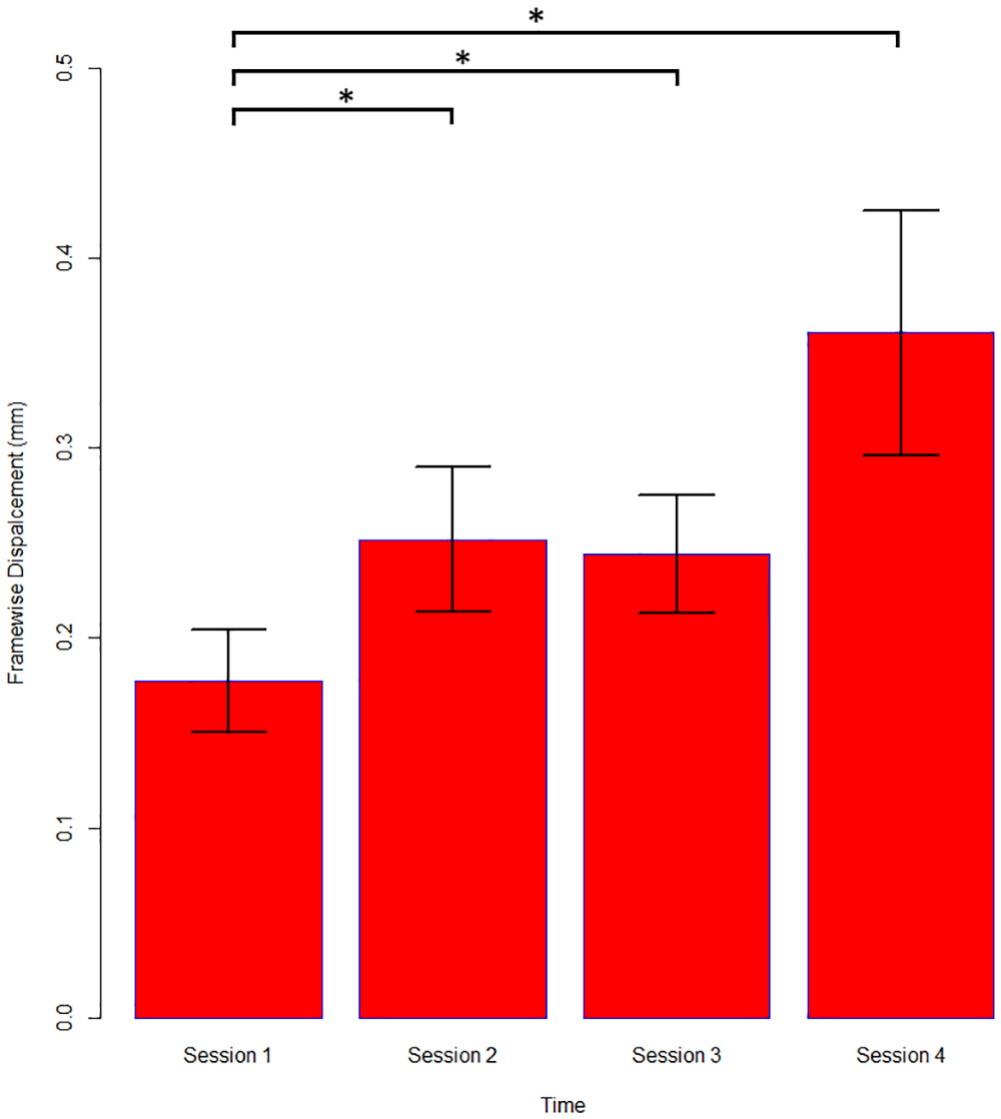
Movement as a function of time. There is an increase of movement of approximately 40% in the second and third session and of 100% in the fourth session relative to the first. The bars represent the mean in-scanner movement for each session across patients and controls irrespective of the effect of video and age. The standard error is represented by T bars. Amount of movement for sessions 2–3 and 4 were significantly greater compared to session 1 (p< 0.05). Significant differences marked with asterisk.

The amount of movement inside the scanner was greater for sessions 2-3-4 relative to session 1, t(176) = 2.54, 2.72, 4.34 respectively (p<0.05 for all) (Fig 2). Older subjects moved less than younger subjects shown by a significant effect of age, t(64) = -2.49, p <0.05. The effect of movie on in-scanner movement was not statistically significant when it is considered across all sessions. However, the movie significantly reduced movement when played in the first two sessions, as evidenced by the significant movie—time interaction in the session 2< session 1 contrast, t(176) = -2.495, p <0.05. This amounted to an average reduction in movement of approximately 40% by playing the movie in the first 2 sessions.

### 3.3 Effect of the movie on IED and EEG-fMRI maps

Eighty percent of the patients (38/46) had spikes during the EEG-fMRI scan. For those patients that did not have detectable epileptic activity during the scan, it was absent for all sessions and independent of session type (movie or rest).

The mean number of IED per session across all patients and sessions was 112.4 (standard deviation 88). Patients had between 1 and 4 types of IED with 55% of patients having 1 type, 29% having two types and 13% having 3. Only one patient had 4 different types of IED.

**Fig 3:**
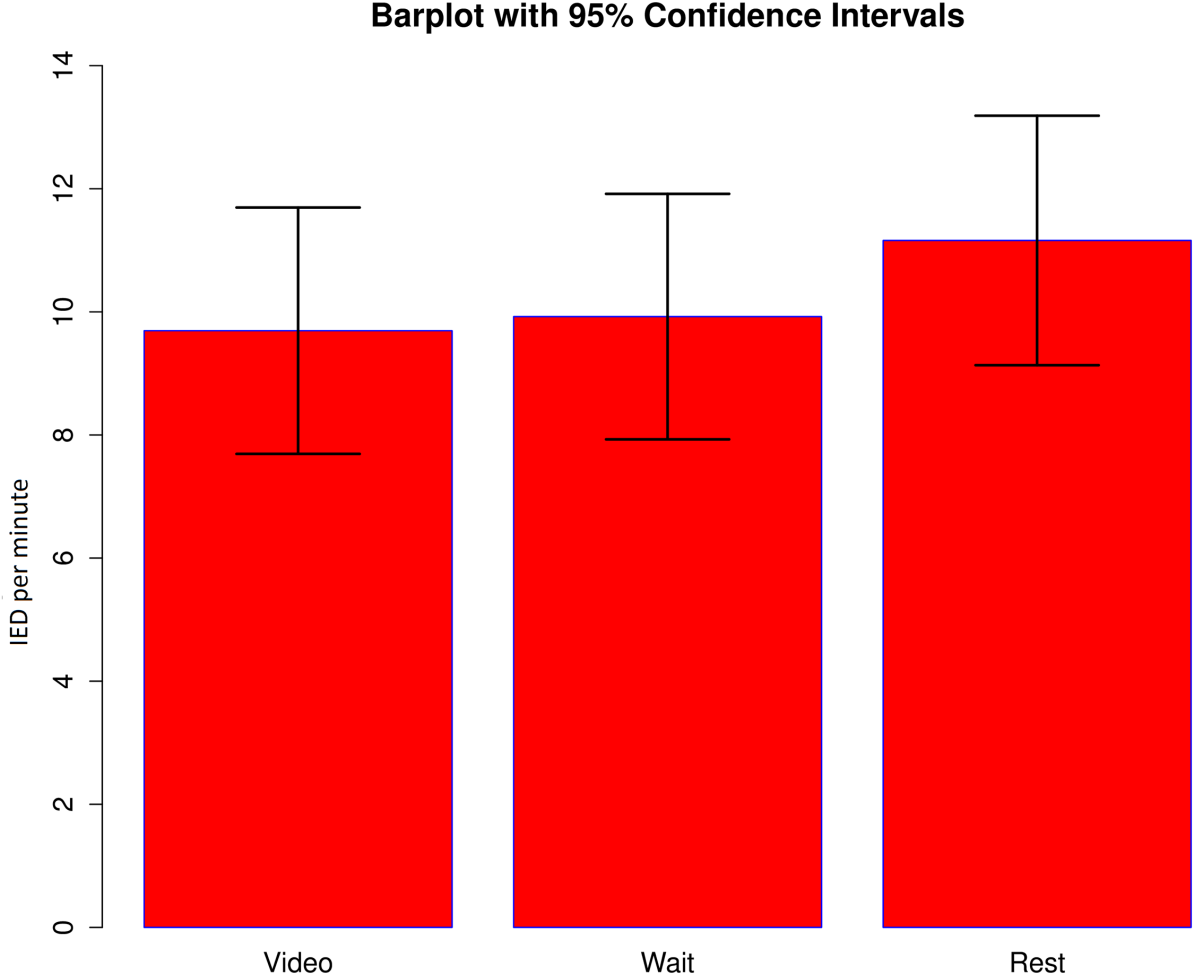
Rate of interictal epileptiform discharges (IED). Bar represent mean number of IED per minute during the resting state sessions and during the two conditions of the video session (video clip and “please wait” screen). There is no significant difference between the rates of IED in these three conditions.

Using an ANOVA it was found that there was no statistically significant effect of condition (video, please wait, rest) on IED rate, F(2,226) = 0.273, p>0.05 (Fig 3).

There was no significant effect of movie stimuli on the concordance of EEG-fMRI maps: the proportion of concordant maps derived from sessions with the movie stimuli did not significantly differ from the proportion of concordant maps derived from resting sessions (χ^2^ (1, N = 137) = 0.81, p>0.05)

### 3.4 Effect of scan duration on EEG-fMRI map concordance

The EEG-fMRI maps for 18 out of 28 (64%) patients who completed the 4 sessions were classified as concordant. When considering the first 20 minutes of scanning alone (2 sessions), the proportion of concordant maps was very similar 63% (24/38). This was despite the potential increase in age and compliance of the group who could tolerate 4 sessions and the increase in available data.

**Fig 4:**
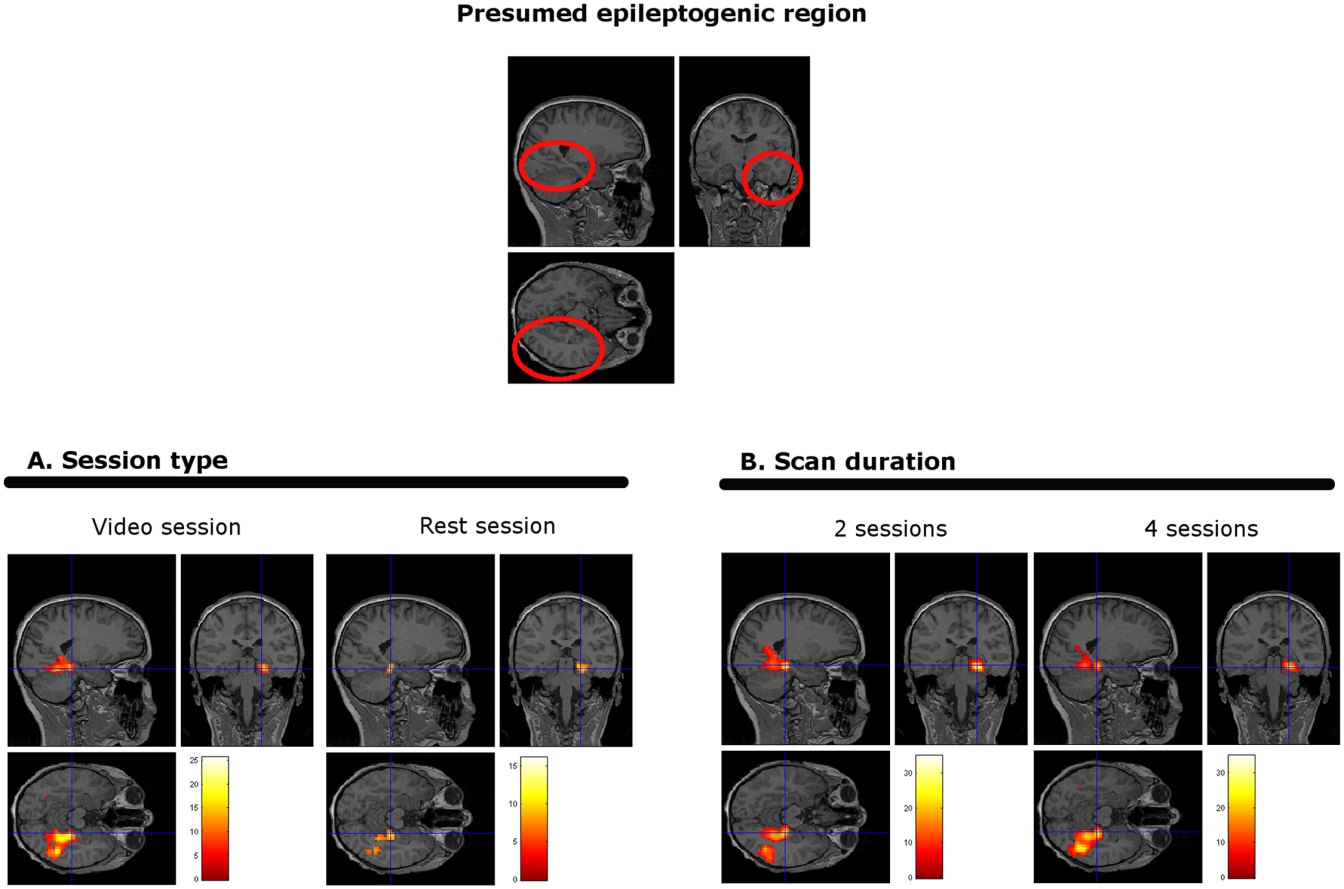
Example of IED related EEG-fMRI maps. Patient with epilepsy #30. Clinical information: 11 years old male; presumed epileptogenic region located in the right posterior quadrant (circled in red). No structural lesion. Interictal and ictal epileptic activity arising from right posterior quadrant. Seizure semiology lateralizes to the right with involvement of the temporal lobe. EEG-fMRI showed significant BOLD signal changes in the posterior hippocampus, para hippocampal gyrus and fusiform gyrus. Section A shows an example of the activity in the presumed epileptogenic region during video and rest sessions. Section B shows an example of the activity in the presumed epileptogenic region during 2 sessions versus 4 sessions.

Fig 4 (Fig 4) illustrates an example of BOLD changes in the epileptogenic region during the different type of sessions and for different scan durations

### 3.5 Effect of design efficiency on EEG-fMRI map concordance

The classification accuracy of the logistic regression of design efficiency on EEG-fMRI concordance was 65%. This was found to be a statistically significant effect (Z = 2.925, p<0.05).

The classification accuracy of the logistic regression model investigating the factors that predict concordance had a classification accuracy of 65%. The effect of motion, as measured by mean framewise displacement, was found not to be statistically significant (Z = -1.43, p>.05). TSNR was did not predict concordance either (Z = -.453,p>.05). However, design efficiency was a statistically significant predictor of concordance (Z = 2.49,p < .05).

## Discussion

EEG-fMRI studies in epilepsy have been much more frequently performed in adult patients compared to children. The greater challenges of scanning children may in part explain this difference. However the potential advantages of well targeted surgical intervention in children make it important to develop strategies that facilitate brain imaging techniques in this population. In addition, tolerable and minimally invasive tests with reduced health risks (i.e. those associated with ionising radiation or anaesthesia) are particularly important in children.

In this study, a more child-friendly approach to EEG-fMRI was implemented and assessed in children with epilepsy in the age range 6–18 without using sedation which is one of the largest cohorts of children whom have undergone EEG-fMRI scans. To our knowledge, this is the first EEG-fMRI study in epilepsy using stimulation during the recording.

Sedation has been the approach of choice in paediatric EEG-fMRI studies [22]. Specific drugs such as chloral hydrate have shown minimal effects on IED [31], and on the sensitivity of EEG-fMRI to map the epileptogenic regions [21]. However the effects of these drugs on the BOLD signal [32, 33] and on resting state networks [34] cannot be ignored as a potential confound. The results presented here show that good quality EEG-fMRI data can be acquired in children as young as 6 years without sedation.

We recorded a high rate of tolerance of the protocol among our population and we achieved longer scanning durations compared to the average resting state (5–10 minutes) [35, 36] and EEG-fMRI studies (20 minutes) [22] in paediatric subjects. The study was designed so that every subject underwent both type of sessions (movie and rest), therefore good tolerance cannot be solely attributed to the movie. In some individuals it was necessary to start with the movie to enable the scan to proceed, and it was shown to reduce motion (in the first 2 sessions) demonstrating that it significantly contributed to compliance/tolerability.

EEG-fMRI is typically recorded in resting state with eyes closed [4] under the assumption that IED are more likely during this state. We showed IED rate was not affected by the movie (Fig 3) and neither were the concordance of the results obtained. IED frequency changes resulting from cognitive tasks is variable among subjects [37] which is in keeping with our findings. The movie clips were chosen to suit a broad age spectrum, with no verbal content in order to avoid involvement of specific language areas Our results show that this type of stimulation can be used in EEG-fMRI epilepsy studies without affecting the IED and the BOLD signal changes in the epileptogenic regions.

In addition to showing that there was no penalty for increasing the tolerability of EEG-fMRI for children this opens the door to the possibility that EEG-fMRI studies during stimulus could be effective for localisation of focal epilepsy in tandem with cognitive fMRI that is frequently obtained for motor and language mapping [38] in this patient group.

We demonstrated that design efficiency is predictive of EEG-fMRI concordance, this suggest that not only the rate but the combination of rate and the distribution in which IED occur is important for finding significant results. To our knowledge no EEG-fMRI study of patients with epilepsy has examined the effect of design efficiency on the quality of the EEG-fMRI result. Design efficiency of the EEG-fMRI experiment provides a measure of the power of the test to map the epileptogenic region, therefore it is recommended that EEG-fMRI studies in patients with epilepsy measure their design efficiencies and interpret and report their results in light of them.

The results presented here represent the first systematic attempt to address the optimal scan duration. Typical scan times in adult EEG-fMRI studies range from 40 to 90 minutes in order to maximize the chances of capturing IED inside the scanner [6, 9]. While longer scanning times result in more data and events this does not necessarily result in improved sensitivity and the later sessions are associated with greater subject movement. Irrespective of the effects of the movie and age, we can predict from the model an increase in movement of approximately 40% in session 2 and 3 and of 100% in session 4 relative to the first session (Fig 2 with individual examples shown in S1 Fig). We also observed an interaction between scan duration and watching the movie regardless of age. While it is an effective strategy to reduce movement levels in the first two sessions it did not make any significant improvement in the later sessions. Taken together with the concordance results that showed no difference between 20 and 40 minutes recordings in the ability to map the epileptogenic region, and given that IED in this population are unlikely to be captured in later sessions if they are not present in the first session our data suggest that EEG-fMRI for approximately 20 minutes may be the optimal scanning length in unsedated children.

We acknowledge several limitations to this study.

Firstly, our results may be limited by the accuracy of the measure chosen to classify concordance of EEG-fMRI maps. The epileptogenic region was defined by the presurgical tests as opposed to the surgical outcome which is considered the gold standard to define this region. Therefore it was not possible to assess the true positive or negative predictive value of the EEG-fMRI results. The concordance measure used however provides a metric that allowed the objective comparison of the different factors that contribute to the ability of EEG-fMRI to map IEDs.

Secondly, EEG-fMRI studies in epilepsy may be limited by the hemodynamic response models used. While there is not a consensus about the best hemodynamic response model to use in these studies [39], the canonical HRF and its derivatives have a degree of flexibility and have been consistently shown to capture IED-related BOLD signal changes in the epileptogenic regions in adult [4] and paediatric populations [21]. In general, broadly canonical responses have been found at the presumed site of the epileptic focus [40]. Despite the potential limitations of the HRF model used, it should not affect the relationship between concordance and the various factors examined. As the same hemodynamic model was used throughout this should not have influenced the conclusions of the study.

Lastly, the conclusions about the optimal duration of EEG-fMRI can be extrapolated to a subpopulation of patients with frequent spikes such as the one recruited in this study. In subjects with infrequent activity longer scanning times may be optimal.

## Conclusions

In conclusion our data shows that good quality EEG-fMRI data can be obtained in children as small as six without sedation. Child friendly stimulus such as movies improve the tolerability of the test and reduce the movement inside the scanner without having a significant effect on the epileptic activity and/or the EEG-fMRI maps.

Twenty minutes of EEG-fMRI acquisition seem to be an optimal duration that maximizes the likelihood for obtaining significant results and to minimize in-scanner movement in our patient group with frequent IEDs.

Design efficacy derived from the occurrence of interictal epileptiform discharges is a relevant factor in the quality of EEG-fMRI maps, the quantification of this factor may help in the interpretation of EEG-fMRI results.

## Supporting Information

S1 Fig Representative examples of inside-scanner movement and its correlation with video sessions. Five examples of individual movement parameters across the different scan sessions. Blue, red and green lines represent the rotation (y axes) on the three axes in space across time/images (x axes) relative to the first image. Each session comprises 300 images, different sessions are separated by vertical dotted lines. For each case the type of session (video/ rest) is represented underneath the X axes by Tom & Jerry picture (movie session) and eyes closes (rest session). A and B are representative of the most common pattern across subjects showing increased movement after 2 sessions and an effect of video in the first/second session. D and E show two examples with large effect of video on the movement. It is even noted increase of movement during the “please wait” screen. C represents a patient who fell sleep after the first session with video.
(TIFF)
