# Supplementary figures and images for: Optimising EEG-fMRI for Localisation of Focal Epilepsy in Children
Source: PLoS One. 2016 Feb 12;11(2):e0149048. doi: 10.1371/journal.pone.0149048 (PMC4752259; doi:10.1371/journal.pone.0149048)

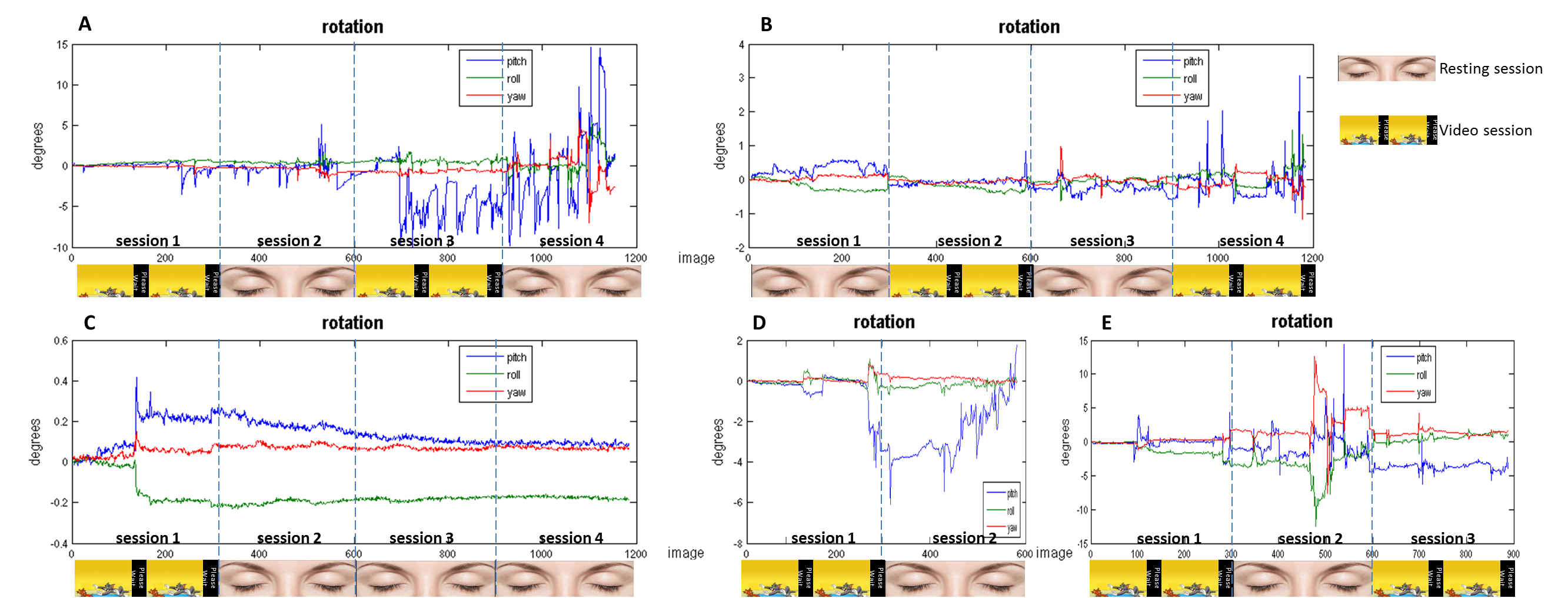

Supplement: S1 Fig — Five examples of individual movement parameters across the different scan sessions. Blue, red and green lines represent the rotation (y axes) on the three axes in space across time/images (x axes) relative to the first image. Each session comprises 300 images, different sessions are separated by vertical dotted lines. For each case the type of session (video/ rest) is represented underneath the X axes by Tom & Jerry picture (movie session) and eyes closes (rest session). A and B are representative of the most common pattern across subjects showing increased movement after 2 sessions and an effect of video in the first/second session. D and E show two examples with large effect of video on the movement. It is even noted increase of movement during the “please wait” screen. C represents a patient who fell sleep after the first session with video. (TIFF) [file pone.0149048.s001.tiff]
